# A Robust Protocol to Isolate Outer Membrane Vesicles from Nontypeable *Haemophilus influenzae*

**DOI:** 10.3390/mps6020042

**Published:** 2023-04-07

**Authors:** M. Daben J. Libardo, Eberhard Durr, Lorraine D. Hernandez

**Affiliations:** Discovery Biology, Infectious Diseases & Vaccines, Merck & Co., Inc., West Point, PA 19486, USA; mark.daben.libardo@merck.com (M.D.J.L.); eberhard.durr@merck.com (E.D.)

**Keywords:** outer membrane vesicles, isolation protocol, nontypeable *Haemophilus influenzae*

## Abstract

Outer membrane vesicles (OMVs) are lipid structures containing various biomolecules in their native environment and are spontaneously shed by gram-negative bacteria. OMVs perform several biological functions critical to both bacterial physiology and pathogenicity. Scientific research on OMV function and biogenesis requires a standardized and robust method of isolating these vesicles from bacterial cultures that reliably provide high-purity OMVs. Herein, we describe an optimized protocol to isolate OMVs from overnight cultures of three different strains of nontypeable *Haemophilus influenzae* (NTHi) for use in different downstream applications. Involving mainly differential centrifugation of the culture supernatant, the procedure described is relatively simple, efficient, and generates high-quality OMV preparations from each strain tested with sufficient yields, while preserving the native outer membrane composition.

## 1. Introduction

Gram-negative bacteria spontaneously shed portions of their outer membrane through spherical nanostructures called outer membrane vesicles (OMVs) during growth. This process is highly conserved as OMV production has been observed in virtually all gram-negative species [1]. OMVs are typically 20–250 nm in diameter and are enriched in outer membrane proteins (OMPs), lipopolysaccharides (LPSs), and phospholipids that together encapsulate periplasmic proteins and peptidoglycan fragments [2]. A fraction of OMVs can be multi-lamellar in nature and, as a result, can sometimes contain relatively low levels of inner membrane and cytoplasmic proteins, as well as DNA and RNA. Because of the ubiquitous nature of OMVs, they are thought to perform functions critical to bacterial physiology. Indeed, OMVs have been found to mediate intra- and inter-species communication (via the transfer of small signaling molecules), horizontal gene transfer, and function in the bacterial stress response that results in the release of toxic or misfolded proteins [1,2]. Pathogenic bacteria also use OMVs to deliver toxins into host cells and as decoys for antimicrobial immune effectors [3].

Interest in OMVs not only lies in basic research but also in the translational sciences as well. There is significant interest in the development of these vesicles as a vaccine platform due to their native topological display of several antigenic proteins and other pathogen-associated molecular patterns (PAMPs) without the risk associated with metabolically active cells [4,5]. The OMV-containing, multicomponent vaccine Bexsero (4CMenB) was approved for use against infections caused by *Neisseria meningitidis* serogroup B and has been estimated to provide protection against 66–91% of meningococcal group B strains worldwide [6,7]. Several OMV-based vaccines are currently being developed to target other bacterial pathogens, including *Klebsiella* [8], *Salmonella* [9], *Shigella* [10], *Vibrio cholerae* [11], and *Mycobacterium tuberculosis* [12]. Of note, is the extensive literature describing OMV-based vaccines against nontypeable *Haemophilus influenzae* (NTHi). Several studies have shown that OMVs isolated from NTHi are highly immunogenic and are able to elicit bactericidal antibodies against homologous and heterologous strains offering protection in mouse and chinchilla models of infection [13,14,15,16,17]. These studies lay the groundwork for developing OMV-based vaccines against diseases caused by NTHi, such as chronic obstructive pulmonary disorder (COPD).

As with other gram-negative bacteria, a few methods have been developed to isolate OMVs from NTHi, with each method generating OMVs of varying composition. A widely used method is the ultracentrifugation of the culture supernatant to isolate what are called native OMVs (nOMVs). This method isolates all vesicles (nOMVs) that were spontaneously released to the spent growth medium by the growing and lysed bacteria [7,14,18]. Ultracentrifugation is largely regarded as the least disruptive in terms of preserving not only the molecular composition of the OMVs but also the native conformation of the proteins. Another common method employs ultradiafiltration, which involves passing the bacterial supernatant through a membrane with an appropriate cut-off to remove cellular debris and large aggregates. While effective at isolating OMVs and removing other contaminating microscopic particles, this method requires expert handling of the filtration device and expensive consumables. It has also been reported that ultrafiltration results in the enrichment of smaller-sized vesicles compared to the ultracentrifugation method [19] and, therefore, may not fully recapitulate the diversity of OMVs released by the bacteria under study. OMVs can also be purified from bacterial pellets via detergent treatment typically using deoxycholate to generate vesicles referred to as detergent extracted OMVs (dOMVs) [7]. The endotoxic LPS or lipooligosaccharide (LOS) remains soluble in the detergent solution while the partially soluble remnants of the outer membrane reform vesicles. While advantageous in terms of significantly reducing endotoxicity of the resulting vesicles, dOMVs typically contain less outer membrane proteins with a marked loss in lipoproteins [4,7].

Regardless of the downstream use of OMVs, a simple and efficient method to isolate these vesicles from bacterial cultures is required. Herein, we present a standardized protocol to isolate nOMVs (hereafter referred to simply as OMVs) from overnight cultures of NTHi. We demonstrate the broad applicability of this method by using it to isolate OMVs from strains representing three different NTHi clades [20]. This protocol is the method of choice by several groups developing OMV-based vaccines against NTHi due to its simplicity and the fact that protein antigens are preserved in their native environment and conformation. Furthermore, because the protocol does not require niche equipment and materials, it can be used by scientists who are starting to explore the biology of bacterial OMVs. We show here that this protocol results in a highly purified OMV preparation from each strain tested with sufficient vesicle yield for several downstream studies (e.g., microscopy, proteomics, lipidomics, and in vivo applications). Furthermore, this method, while optimized for NTHi, can also be adapted to isolate OMVs from other gram-negative bacteria as well.

## 2. Experimental Design

Overnight, saturated NTHi cultures are spun down and the bacterial pellet is discarded. The supernatant, containing OMVs, is concentrated to a low volume and the vesicles are then pelleted via ultracentrifugation. The OMV pellet is washed with buffer to remove adherent soluble proteins and finally resuspended in the desired buffer. We demonstrate the robustness of this protocol by isolating OMVs from three different NTHi strains: ATCC53600, 86-028NP, and NTHi 5. We calculate the yield based on the initial culture volume and OD_600_ at the time of harvest. We also show the protein profile of the OMV preparations via SDS-PAGE and check vesicular morphology via transmission electron microscopy (TEM).

### 2.1. Materials

- Nontypeable *Haemophilus influenzae* strains ATCC53600, 86-028NP, and NTHi 5
- Chocolate Agar Plates (Teknova, Hollister, CA, USA, cat no. C4900)
- Brain Heart Infusion broth (BHI, Teknova, Hollister, CA, USA, cat no. B9993)
- Fildes Enrichment (ThermoFisher Scientific, Waltham, MA, USA, cat no. R45037)
- Nicotinamide Adenine Dinucleotide (NAD, Millipore Sigma, St. Louis, MO, USA, cat no. 10127990001)
- Sterile 250 mL Polycarbonate Culture Flask (ThermoFisher Scientific, Waltham, MA, USA, cat no. PBV250)
- Two hundred fifty milliliter Nalgene Centrifuge Bottle (ThermoFisher Scientific, Waltham, MA, USA, cat no. 3141-0250PK)
- Sterile 150 mL Stericup Vacuum Filtration System, 0.22 μm, PVDF (Millipore Sigma, St. Louis, MO, USA, cat no. S2GVU01RE)
- Centricon Centrifugal Filter 100 kDa MWCO (Millipore Sigma, St. Louis, MO, USA, cat no. UFC710008)
- Three milliliter Thickwall Polycarbonate Tube, Beckman Coulter (ThermoFisher Scientific, Waltham, MA, USA, cat no. 362305)
- 1X Gibco Dulbecco’s Phosphate Buffered Saline (DPBS, ThermoFisher Scientific, Waltham, MA, USA, cat no. 14190144)
- Sterile 1 mL BD Insulin Syringes, 27 5/8 G (ThermoFisher Scientific, Waltham, MA, USA, cat no. 329412)
- Sterile BD 21G × 2 mm needle (ThermoFisher Scientific, Waltham, MA, USA, cat no. 305129)
- Sterile 3 mL Henke-Ject Luer Lock Syringe (ThermoFisher Scientific, Waltham, MA, USA, cat no. 4020-X00V0)
- Durapore 0.45 μm PVDF Syringe Filter (Millipore Sigma, St. Louis, MO, USA, cat no. SLHV013SL)
- Pierce Micro BCA Assay Kit (ThermoFisher Scientific, Waltham, MA, USA, cat no. 23235)
- Corning 96-well Microtiter Plate (ThermoFisher Scientific, Waltham, MA, USA, cat no. 07-200-89)
- Novex 4–20% Tris-Glycine WedgeWell^TM^ Precast Gels (ThermoFisher Scientific, Waltham, MA, USA, cat no. XP04202BOX)
- Novex 10X Tris-Glycine SDS Running Buffer (ThermoFisher Scientific, Waltham, MA, USA, cat no. LC2675)
- Novex 2X Tris-Glycine SDS Sample Buffer (ThermoFisher Scientific, Waltham, MA, USA, cat no. LC2676)
- NuPAGE 10X Sample Reducing Agent (ThermoFisher Scientific, Waltham, MA, USA, cat no. NP0009)
- PageRuler Plus Prestained Protein Ladder (ThermoFisher Scientific, Waltham, MA, USA, cat no. 26619)
- InstantBlue Coomassie Stain (ThermoFisher Scientific, Waltham, MA, USA, cat no. ISB1L)
- Formvar/Carbon Supported 300 mesh Copper Grid (Millipore Sigma, St. Louis, MO, USA, cat no. TEM-FCF300CU50)
- Phosphotungstic Acid Hydrate (Millipore Sigma, St. Louis, MO, USA, cat no. 79690)
- Uranyl Acetate Dihydrate (ThermoFisher Scientific, Waltham, MA, USA, cat no. 18-607-644)
- Bovine Serum Albumin (BSA, Millipore Sigma, St. Louis, MO, USA, cat no. 10711454001)

### 2.2. Equipment

- 37 °C, 5% CO_2_ incubator with shaker
- SpectraMax M3 Plate Reader
- Beckman Coulter Avanti J-20 XP centrifuge with a JLA-16.250 rotor
- Sorvall Legend RT Tabletop Centrifuge
- Beckman Coulter Optima TLX Ultracentrifuge with a TLA100.4 rotor
- XCell SureLock Gel Electrophoresis Tank
- Electrophoresis Power Supply
- Platform Shaker
- BioRad ChemiDoc MP Gel Imager
- FEI Tecnai Spirit Twin Transmission Electron Microscope

## 3. Procedure

### 3.1. Isolation of Outer Membrane Vesicles (OMVs)

1. Streak out NTHi strains onto separate chocolate agar plates and incubate overnight in a 37 °C, 5% CO_2_ incubator.
2. Pre-warm 150 mL of NTHi growth medium (BHI broth + 2% Fildes enrichment + 20 μg/mL NAD) in 250 mL vented shake flask to 37 °C.
3. Inoculate pre-warmed media with 3–5 isolated colonies of NTHi from step 1 and incubate overnight in a 37 °C, 5% CO_2_ incubator, shaking at 250 rpm.
4. To obtain a measure of culture density, prepare a 1:10 dilution of the overnight culture by mixing 100 μL of the overnight culture and 900 μL fresh growth medium. Measure the OD_600_ of the resulting suspension. Multiply the OD_600_ by 10 to account for the 1:10 dilution.
5. Transfer the remainder of the overnight culture to a 250 mL centrifuge bottle and pellet the cells at 10,000× *g* for 10 min at 4 °C. Carefully remove the supernatant and discard the bacterial pellet.
6. Filter the supernatant using a 0.22 μm PVDF Stericup vacuum filter flask and collect the filtrate.
7. To concentrate the filtered supernatant, use 2 Centricon centrifugal filters loading 60 mL of supernatant into each filter. Discard the remaining ~30 mL of supernatant (only 120 mL of the supernatant will be used for OMV isolation). The total volume of the supernatant can be scaled up by increasing the initial overnight culture as needed based on the yield obtained from initial trials.
8. Centrifuge the filter assemblies at 3500× *g* for 20 min at 4 °C in a Sorvall Legend RT tabletop centrifuge using a swinging bucket rotor. Other NTHi strains or corresponding isogenic mutants may require longer run times for supernatant concentration. In each case, extend run time in 10 min increments.
9. When the retentate in the Centricon filter reaches a volume of ~2 mL, elute the concentrated culture supernatant using the Centricon collection cup. Spin down the assembly at 1000× *g* for 1 min at 4 °C. Note that at this point, the concentrated supernatant will be slightly more viscous and visibly darker due to the concentration of the Fildes enrichment.
10. Transfer the concentrated supernatant containing the OMVs into 3 mL thick-walled polycarbonate tubes and add cold 1X DPBS until the total volume is ~3 mL.
11. To check the sterility of the OMV prep, spread a 50 μL aliquot onto a fresh chocolate agar plate. Incubate the plate overnight at 37 °C, 5% CO_2_. If colonies grow on the plate overnight, discard the entire OMV suspension as it contains contaminating viable bacterial cells.
12. Pellet the OMVs by centrifuging tubes at 135,000× *g* (~50,000 rpm) for 18 h (overnight) at 4 °C in the Optima TLX ultracentrifuge using a TLA100.4 rotor. If time permits, this step can be reduced to a minimum of 5 h without compromising the yield. If there are NTHi colonies in the agar plates from Step 11, stop here and discard the OMV pellet, otherwise, proceed to Step 13.
13. Carefully remove as much of the supernatant as possible using a 1 mL pipette. Once the OMV pellet is visible, photographs can be taken against a white background if desired.
14. Fully resuspend the OMV pellet in 1 mL of ice-cold 1X DPBS by pipetting up and down several times until no visible clumps can be observed. Add 2 mL of fresh ice-cold 1X DPBS. Vortex the tubes at max speed for 30 s. This step washes away the soluble proteins that may be loosely adsorbed to the OMV surface. More importantly, this also washes away most of the remaining Fildes enrichment associated with the OMVs.
15. Pellet the OMVs by centrifuging at 135,000× *g* for 5 h at 4 °C using the Optima TLX ultracentrifuge using the TLA100.4 rotor.
16. Carefully remove as much of the supernatant as possible leaving only a wet OMV pellet.
17. Resuspend the OMV pellet in 1 mL of ice-cold 1X DPBS until no visible clumps can be observed. This volume can be increased to 2–3 mL depending on how much starter culture was used. This volume is called V_res_ and will be used later to calculate OMV yield.
18. To further resuspend the OMVs and make sure that no macroscopic OMV aggregates remain, pass the OMV suspension through a 1 cc Insulin syringe three times and vortex the suspension at max speed for 30 s.
19. Pass the OMV suspension three times through a 21G × 2 mm needle attached to a 3 cc luer-lock syringe making sure to collect the OMV suspension inside the syringe after the third pass. This step further ensures that any small OMV aggregates are broken up.
20. Aspirate ~1 mL of air to have an air pocket between the needle and the OMV suspension inside the syringe.
21. While the OMV suspension is inside the syringe, carefully remove the needle and replace it with a 0.45 μm PVDF syringe filter.
22. Pass the OMV suspension through the syringe filter collecting the final suspension in a sterile 1.5 mL tube.
23. Divide the OMV suspension into 250 μL aliquots (or desired volume) and freeze at −80 °C.

### 3.2. Quantification of Protein Content and Calculation of OMV Yield

1. Thaw an aliquot of the OMV suspension on ice for ~30 min. (Any future use of the OMVs should be preceded by the thawing of the frozen suspension on ice.)
2. When measuring the protein concentration of the OMVs, prepare a dilution series of the OMVs to account for the possibility of the OMV protein concentration exceeding the upper limits of the calibration curve. Typically, a 1:4 starting dilution is necessary.
3. Measure the protein concentration of the OMV prep using the Pierce MicroBCA Assay Kit according to the manufacturer’s protocol and using the accompanying bovine serum albumin (BSA) as a standard.
4. Perform the MicroBCA assay in a 96-well polystyrene microtiter plate and read the absorbance at 562 nm using a standard plate reader.
5. Calculate the protein concentration of the OMV suspension using the calibration curve obtained in the MicroBCA Assay and, if necessary, account for any dilutions done previously.
6. The OMV yield (per 120 mL culture supernatant) is calculated based on the obtained protein concentration and the V_res_ normalized against the OD_600_ of the starting culture using the following equations:

$$\mu g\;protein=OMV\;Protein\;conc\;\left(\mu g/mL\right)\times V_{res}\;\left(mL\right)$$

$$OMV\;Yield\;\left(per\;120\;mL\;supernatant\right)=\frac{\mu g\;protein}{OD_{600}\;starter\;culture}$$

### 3.3. SDS-PAGE Analysis of OMV Protein Profile

Depending on the resulting OMV protein concentration, load between 5 and 30 μg protein per lane in an SDS-PAGE gel. Novex Tris-Glycine WedgeWell^™^ gels hold up to 60 μL of sample/well making it feasible to load enough protein for dilute OMV suspensions. For illustration purposes, in the following steps, the OMV suspension is diluted to 430 μg/mL, resulting in a final protein loading of ~10 μg per lane. The sample can be adjusted as needed based on the obtained OMV concentration.

1. Prepare 56 μL of each OMV suspension at 430 μg/mL in 1X DPBS.
2. Add 70 μL of 2X Tris-Glycine SDS Sample Buffer and 14 μL of 10X NuPAGE Sample Reducing Agent to the OMV samples.
3. Split the mixture into two 70 μL aliquots labeling one “heated” and the other “unheated”. Boil the “heated” fraction at 100 °C for 10 min and allow to cool to room temperature.
4. Load 50 μL of each “heated” and “unheated” sample into separate wells of a Novex 4–20% Tris-Glycine WedgeWell^™^ gel. Run the gel at a constant 225 V for 45 min using 1X Tris-Glycine SDS Running Buffer.
5. Remove the gel carefully from the cassette and stain using InstantBlue Coomassie Stain overnight in a low-speed shaker at room temperature.
6. Image the gel using a BioRad ChemiDoc MP Gel Imager or any similar gel visualization device.

### 3.4. Transmission Electron Microscopy (TEM) of OMVs

1. Secure 300 mesh copper grids with forceps and clasp tightly. Rinse the grids with 0.01% BSA and wick off any excess solution using a filter paper.
2. Place a 2 μL aliquot of each OMV suspension (several dilutions may be required to ensure optimum vesicle population in every field of view) onto each copper grid and allow to air dry for 10 min. Wick off any residual material in the copper grid using a filter paper.
3. Stain the samples with 20 μL of 2% Phosphotungstic acid or 2% Uranyl Acetate and incubate grids for 60 s at room temperature. Remove any excess solution using a clean filter paper.
4. Mount the copper grids onto an FEI Tecnai Spirit Twin Transmission Electron Microscope and examine samples at high magnification at 80 kV.
5. Take multiple images throughout the grid. Measure the diameter of several OMVs using the pixel-to-micron conversion factor specific to the microscope used.

## 4. Expected Results

**Figure 1:**
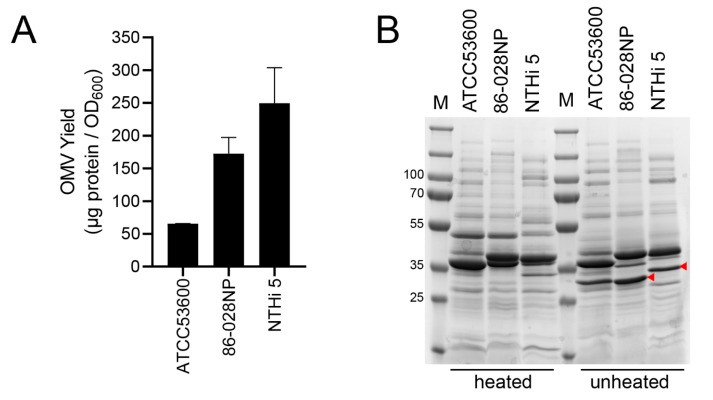
(**A**) OMV yield as measured by protein concentration obtained from three different NTHi strains using the protocol described above. Bars represent Mean ± SEM, *n* = 3. (**B**) Tris-Glycine SDS-PAGE analysis of the protein profile of OMVs isolated from the indicated NTHi strain. Heated samples were boiled at 100 °C for 10 min, while unheated samples remained at room temperature. A total of ~10 μg protein was loaded onto each well. Red arrowheads indicate the position of the heat-modifiable OmpP5.

We find that a 150 mL overnight culture inoculated with 3–5 colonies of NTHi routinely results in an OD_600_ between 1.1 and 1.9. The supernatant from this culture typically yields between 65 and 550 μg of protein which translates to 60–300 μg/OD_600_ of OMVs. Note that in this context, the OMV concentration is the concentration of OMV-associated proteins in the suspension, rather than the actual concentration of vesicles. Figure 1A shows the OMV yield we obtained from three different NTHi strains across three isolation batches. We find that the amount of OMVs isolated is dependent on several factors, chief among which is the NTHi strain being used. The variability apparent in Figure 1A is due to the fact that the strains used grew at varying rates to different densities and were harvested at widely different ODs (ATCC53600, 86-028NP, and NTHi 5 at average OD_600_ values of ~1.2, ~1.4, and ~1.7, respectively). All other factors being equal, one can expect to isolate more vesicles from a denser culture. Despite the significant interstrain variability that exists in NTHi, the protocol presented here is broadly applicable to OMV isolations from NTHi strains that belong to different clades: ATCC53600 (Clade II), NTHi 5 (Clade IV), and 86-028NP (Clade V) [20]. Depending on the yield of the initial trials and the requirement for higher OMV concentrations, the culture volume can be scaled up to 1L to obtain OMV yields of 300–900 μg/OD_600_.

During several rounds of optimizing the protocol presented herein, we found that the OMV yield was optimal when initial ultracentrifugation of the concentrated supernatant was performed at 135,000× *g* overnight (18 h). Other protocols spin at similar speeds or higher for 1 to 4 h [14,15]. In our hands, however, when ultracentrifugation was done for 2 h or less, even at higher speeds, the yield was typically less than half of what we reported here. We, therefore, decided with an overnight spin to initially pellet OMVs to maximize yield and introduce a good stopping point in the protocol. The latter reason is particularly important in avoiding extended laboratory working hours when starting with a larger culture volume (e.g., 1 L) that requires a longer time for preconcentrating the supernatant. In other OMV isolation protocols, the first centrifugation step resulting in an OMV pellet from the culture supernatant is followed by a density gradient centrifugation step or purification using gel filtration. This extra step washes away any proteins that may be adsorbed to the OMV surface and separates any soluble contaminants from the vesicles. While effective at removing contaminating molecules, these steps require additional reagents, materials, and user experience. The protocol we present here shows that washing the OMV pellet via simple resuspension in a buffer is a good alternative for simple and non-stringent downstream applications (e.g., morphological analysis via microscopy). However, we note that some applications (i.e., in vivo testing) require a much higher OMV purity, making subsequent purification step(s), such as gel filtration necessary.

After the determination of total OMV protein concentration, the protein profile can be determined via SDS-PAGE analysis. Figure 1B shows the protein profile of NTHi OMVs isolated from three different strains. We find that 5–30 ug of protein per lane gives the best signal-to-noise ratio for OMV proteins of both high and low abundance. It is widely known that outer membrane proteins P2 and P5 make up the majority of proteins in the NTHi outer membrane and OMVs [14,21]. We routinely run heated and unheated OMV samples in the gel to ensure the fidelity of the proteins, as represented by the heat lability of P5. Consistent with previous observations [13,14], the ~27-kDa OmpP5 protein migrates to a higher MW region (closer to what is predicted by the gene sequence) upon heating as can be markedly seen in Figure 1B (red arrowheads). The protein profile obtained from SDS-PAGE analysis can be used to compare OMVs isolated from the same strain but at different times. In our hands, the protocol presented here is robust enough to deliver highly reproducible protein profiles even for OMVs that were isolated years apart from each other.

**Figure 2:**
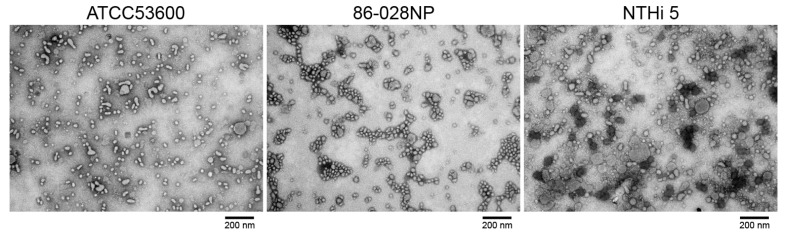
Transmission electron micrographs (TEM) images of OMVs isolated from three different NTHi strains as indicated. Images are taken at 67,000× magnification.

As a final qualitative measure of OMV fidelity, we routinely image the OMVs via TEM. As shown in Figure 2, the OMVs isolated using this protocol exhibit morphologies typical of native and intact OMVs. The vesicles appear spherical (or roughly spherical) and mostly exist either as single isolated structures or in small aggregates. We measured the diameter of 20–50 vesicles in the TEM images and used the pixel-to-micron conversion factor to determine the actual OMV diameter. The size distribution of the OMVs is relatively narrow with vesicles measuring between 15 and 100 nm. Little morphological differences can be seen in OMVs isolated from the three different strains used in this study. Finally, there is no visual evidence of protein-like structures and/or contaminating microorganisms in any of the images obtained, demonstrating the high quality and purity of the OMV suspension obtained using the protocol detailed here.

The protocol presented here can be easily adapted to isolate OMVs from other gram-negative bacteria taking into account several key differences. First, NTHi grown in standard laboratory media are non-motile and do not express flagella and only a few strains express pili-like structures [22]. Therefore, the OMV prep from NTHi cannot be contaminated with these appendages. For other gram-negative bacteria, however, dissociated flagella or pili must be removed through other means because they tend to pellet with OMVs. A similar contaminating issue is presented by capsular polysaccharides present in many gram-negative species but are absent in NTHi. We envision that additional steps, such as gel filtration, may be necessary when adapting this protocol to other bacteria to obtain purified OMVs.

## 5. Conclusions

We present here a simple, efficient, and robust method to isolate OMVs from the culture supernatants of NTHi. The protocol described here is cost-effective and yields high-quality OMVs in amounts sufficient for several downstream applications without compromising molecular composition and vesicular morphology, making it the preferred methodology for NTHi OMV preparation. We note that for downstream applications that require higher purity or better OMV grades, subsequent purification steps not covered in this report (i.e., density gradient centrifugation and gel filtration) will need to be done. This protocol can also be adapted and modified as needed to isolate OMVs from other gram-negative bacteria.

## Data Availability

Original data are available on request.
